# Fully individualized models for cross-sectional and longitudinal network-based tau spread

**DOI:** 10.1162/IMAG.a.1053

**Published:** 2025-12-09

**Authors:** Christopher A. Brown, Sandhitsu R. Das, John A. Detre, Ilya M. Nasrallah, Paul A. Yushkevich, Corey T. McMillan, David A. Wolk

**Affiliations:** Department of Neurology, University of Pennsylvania, Philadelphia, PA, United States; Department of Radiology, University of Pennsylvania, Philadelphia, PA, United States

**Keywords:** diffusion MRI, structural connectivity, Alzheimer’s disease, tau PET, heterogeneity

## Abstract

Heterogeneity in regional tau burden limits evaluation of disease progression using one-size-fits-all approaches. Prior work using tau positron emission tomography (PET) has highlighted the important role of connectivity to epicenters of tau pathology in explaining this heterogeneity. However, previous studies using population-based epicenters or connectomes fall short of a fully individualized approach to predicting regional tau burden. We use diffusion MRI-derived structural connectomes to assess the prediction of regional tau burden using distance along individual structural connectomes from individualized epicenters of tau pathology both cross-sectionally and longitudinally. Fully individualized models of connectivity and epicenters outperformed models using either population-based connectomes or epicenters in explanation of cross-sectional and longitudinal regional tau burden, improved prediction in validation datasets, and produced stronger single-subject level prediction. Together, these findings demonstrate the power of a fully individualized approach to explain regional tau heterogeneity and provide the strongest *in vivo* evidence to date for network-based spread of tau pathology.

## 1 Introduction

Alzheimer’s disease (AD) is pathologically defined by accumulation of amyloid-β (Aβ) plaques and neurofibrillary tangles (NFTs) composed of tau aggregates (Montine et al., 2012). Advances in molecular positron emission tomography (PET) over the last 20 years have enabled us to assess these pathologies *in vivo*. In particular, amyloid PET imaging has reaffirmed the relatively diffuse and stereotypical pattern of Aβ plaques in those with AD, and tau PET has reinforced that the dominant pattern of pathologic spread is defined by Braak staging with tau pathology starting in the transentorhinal cortex and hippocampus prior to spreading to additional limbic and temporal neocortical regions and then to parietal, frontal, and occipital cortex (Braak & Braak, 1991; Brier et al., 2016; Klunk et al., 2004; Schwarz et al., 2016). However, tau PET has additionally uncovered heterogeneous patterns of tau deposition with evidence of consistent deviations from this canonical pattern and a large degree of individual variability in the sequencing and timing of tau propagation (Ossenkoppele et al., 2016; Vogel et al., 2021; Xia et al., 2017). These inter-individual differences are particularly important when considering evaluation of therapeutic response in clinical trials and approved disease-modifying therapies.

Early neuroimaging studies supported the predilection of neurodegenerative pathology for specific brain networks defined by resting-state functional MRI (rs-fMRI), which was further explored in mouse and *in vitro* models that provided evidence of prion-like trans-synaptic spreading of tau pathology among highly connected neurons (Clavaguera et al., 2009; De Calignon et al., 2012; Iba et al., 2013; Lehmann et al., 2013; Seeley et al., 2009). The advent of tau PET led to an increasing number of studies linking tau propagation between functionally connected brain regions, initially using covariance analyses and later using functional (Franzmeier, Neitzel, et al., 2020; Franzmeier et al., 2019; Vogel et al., 2020) or structural (Jacobs et al., 2018; Vogel et al., 2020) connectivity to specific group-level epicenters of tau pathology. While these studies first focused on attempting to explain the canonical spread of tau pathology using either structural or functional connectivity of regions with the entorhinal cortex (ERC) and other early Braak regions, Franzmeier et. al. provided the first explanation of heterogeneous patterns of tau burden using functional connectivity with patient-centered epicenters of tau pathology (Franzmeier, Dewenter, et al., 2020). Replication of these findings longitudinally provided further evidence of regional tau burden overlapping with brain networks in both cases of canonical spread and when using patient-centered approaches that allow for different epicenters across participants (Frontzkowski et al., 2022; Steward et al., 2023).

While these seminal studies provide an important foundation for network-based propagation of tau pathology, they have relied upon population-based connectomes due to the inherent noise in individual-level fMRI data (Noble et al., 2019; Vogel et al., 2023). A fully individualized approach requires both patient-centered epicenters of tau pathology and individual connectomes. In contrast to fMRI, diffusion MRI (dMRI)-derived structural connectomes provide highly reproducible results in individual subjects (Buchanan et al., 2014; Nakuci et al., 2023; Noble et al., 2019) and offer an opportunity to explore fully individualized models of tau spread. These fully individualized models could provide both improved generalizability, single-subject prediction, and offer improved insight into transmission of tau pathology along structural connections, as has previously been hypothesized but not previously evaluated (Vogel et al., 2023). Given the close link between tau, neurodegeneration, and cognition, these improvements in individualized modeling have important implications for monitoring disease progression in clinical trials and in response to approved therapies.

Here, we use data from the Penn Aging Brain Cohort (ABC) and Alzheimer’s Disease Neuroimaging Initiative (ADNI) for participants with both ^18^F-flortaucipir tau PET and high-angular resolution dMRI to test this fully individualized approach. We defined individualized epicenters of tau pathology using Gaussian-mixture models (GMMs) and used probabilistic tractography to calculate structural connectivity and generate participant-level structural connectomes. Then, we tested whether the structural connectivity-based distance from individualized tau epicenters predicted regional tau burden and compared this approach with those using either group connectomes and/or canonical group epicenters. After generating models of structural connectivity-based tau spread in a discovery dataset, we applied these models to a validation cohort. We further evaluated the impact of Aβ-status on these associations and assessed the ability of generated models to predict regional tau burden in Aβ+ participants at the subject level. Finally, we explored each of these models’ prediction of longitudinal accumulation of tau pathology at the group and individual-subject level in Aβ+ participants.

## 2 Methods

### 2.1 Participants

Participants in this study were selected from the Penn Alzheimer’s Disease Research Center (ADRC) ABC study or ADNI based on availability of (1) Multishell dMRI and tau PET collected within 18 months of each other between January 2016 and January 2024, (2) availability of clinical consensus diagnosis, and (3) Aβ status determined by PET visual read (ABC) or SUVR cutoff (ADNI). Written informed consent was obtained from all ABC participants according to the Declaration of Helsinki under a protocol approved by the University of Pennsylvania Institutional Review Board. Information regarding informed consent in ADNI can be found at http://adni.loni.usc.edu. Clinical consensus was determined by annual consensus conference for both ABC and ADNI, and the clinical diagnosis closest to the tau PET date was used for this study. Given significant differences in diagnostic breakdown and racial representation between ADNI and ABC, data were combined and randomly divided into five splits used for k-fold validation keeping a similar balance between amyloid status between folds. Demographics for the full dataset, as well as a subset of Aβ+ individuals with longitudinal tau PET, are provided in Table 1.

**Table 1.** Participant characteristics.

	Cross-Sectional			
	CU	MCI	Dementia	Total
Age (range)	71 (50–90)	71 (56–86)	75 (65–86)	71 (50–90)
Sex (%) F M	108 (68%) 52 (32%)	28 (44%) 35 (56%)	4 (40%) 6 (60%)	140 (60%) 93 (40%)
Race (%) Asian Black Multiple White	2 (1%) 32 (20%) 1 (1%) 125 (78%)	1 (2%) 9 (15%) 2 (3%) 51 (81%)	0 (0%) 3 (30%) 0 (0%) 7 (70%)	3 (1%) 44 (19%) 3 (1%) 183 (79%)
Education (range)	16 (9–20)	16 (12–20)	16 (12–20)	16 (9–20)
Amyloid Positive (%)	37/160 (23%)	39/63 (62%)	9/10 (90%)	85/233 (36%)
Study (%) ABC ADNI	75 (47%) 85 (53%)	7 (11%) 56 (89%)	0 (0%) 10 (100%)	82 (35%) 151 (65%)

	Longitudinal			
	CU	MCI	Dementia	Total
Age (range)	74 (64–90)	72 (60–79)	79 (72–86)	74 (60–90)
Sex (%) F M	17 (81%) 4 (19%)	8 (38%) 13 (62%)	3 (43%) 4 (57%)	28 (57%) 21 (43%)
Race (%) Black Multiple White	0 (0%) 1 (5%) 20 (95%)	0 (0%) 1 (5%) 20 (95%)	2 (29%) 0 (0%) 5 (71%)	2 (4%) 2 (4%) 45 (92%)
Education (range)	17 (12–20)	15 (12–20)	16 (12–20)	16 (12–20)
Amyloid Positive (%)	21/21 (100%)	21/21 (100%)	7/7 (100%)	49/49 (100%)
Study (%) ABC ADNI	0 (0%) 21 (100%)	1 (5%) 20 (95%)	0 (0%) 7 (100%)	1 (2%) 47 (98%)

Mean (range) shown for continuous measures and n (%) shown for categorical measures; CU: cognitively unimpaired, MCI: mild cognitive impairment, F: female, M: male.

A subset of data used in the preparation of this article were obtained from the Alzheimer’s Disease Neuroimaging Initiative (ADNI) database (http://adni.loni.usc.edu). The ADNI was launched in 2003 as a public–private partnership, led by Principal Investigator Michael W. Weiner, MD. The primary goal of ADNI has been to test whether serial magnetic resonance imaging (MRI), positron emission tomography (PET), other biological markers, and clinical and neuropsychological assessment can be combined to measure the progression of mild cognitive impairment (MCI) and early Alzheimer’s disease (AD). For up-to-date information, see www.adni-info.org.

### 2.2 Image acquisition

MRI data were collected on Siemens 3T scanners using 3D magnetization-prepared gradient-echo (MPRAGE) and multi-shell diffusion turbo-spin echo planar imaging. ADNI data were collected across 16 sites in ADNI3 with Siemen’s scanners (https://adni.loni.usc.edu/data-samples/adni-data/neuroimaging/mri/mri-scanner-protocols/). The ABC protocol included T1 MPRAGE with 0.8 mm isotropic voxels [repetition time (TR) = 2400 ms, echo time (TE) = 2.24 ms, inversion time (TI) = 1060 ms, flip angle = 8°] and dMRI with 1.5 mm isotropic voxels [TR = 3027 ms, TE = 82.8 ms, flip angle = 78°] with 15 directions at b = 300, 30 directions at b = 800, and 64 directions at b = 2000, along with 9 b = 0 images. The ADNI protocol included T1 MPRAGE with 1 mm isotropic voxels [TR = 2300 ms, TE = 2.98 ms, TI = 900 ms, flip angle = 9°) and dMRI with 2 mm isotropic voxels [TR = 3400 ms, TE = 71 ms, flip angle = 90°] with 6 directions at b = 500, 48 directions at b = 1000, and 60 directions at b = 2000, along with 13 b = 0 images.

For both ADNI and ABC, tau PET was acquired using six 5-minute frames from 75 to 105 minutes after injection of ^18^F-flortaucipir. Aβ PET was acquired using 5-minute frames from 90 to 110 minutes after injection of ^18^F-florbetaben or from 50 to 70 minutes after injection of ^18^F-florbetapir in both studies. Details of ADNI PET scanners can be found on the ADNI website (https://adni.loni.usc.edu/help-faqs/adni-documentation/).

### 2.3 Image processing

T1-weighted images were bias corrected and skull stripped using Advanced Normalization Tools (ANTs, http://stnava.github.io/ANTs/) prior to cerebellar, cortical, and subcortical parcellation using multi-atlas segmentation with Joint Label fusion using the MICCAI 2012 BrainColor parcellation (Klein & Tourville, 2012; Landman & Warfield, 2012; Wang et al., 2013). The resulting regions were used as ROIs for PET and dMRI analyses described below.

dMRI data were processed using an in-house pipeline that has been described previously (C. Brown et al., 2024). Briefly, denoising, Gibbs-unringing, and ANTs N4-bias correction were carried out using MRtrix3 prior to FSL eddy with outlier replacement for eddy current correction and distortion correction using b0 field maps (Andersson & Sotiropoulos, 2016; Andersson et al., 2003, 2016; Cordero-Grande et al., 2019; Jenkinson et al., 2012; Kellner et al., 2016; Tustison et al., 2010; Tournier et al., 2019). Quality control included visual inspection and eddy QUAD with scans with >3.5% of overall slice outliers, >20% outliers for a single shell (except for b = 500 in ADNI as only 6 values were obtained), and CNR <3.5 SDs from group mean excluded (Bastiani et al., 2019). FSL dtifit was used to generate fractional anisotropy (FA) maps, which were used to register dMRI data to the T1-weighted image using ANTs linear affine registration. BEDPOSTX was run using a three-fiber per-voxel sticks with a range of diffusivities model with GPU acceleration (Behrens et al., 2003; Hernández et al., 2013). The resulting fiber model was input to PROBTRACKX2 in network mode with modified Euler streamlining, distance correction, 5000 samples per voxel with maximum step-length of 2000 (step length = 0.5 mm), curvature threshold of 0.2, and fiber threshold of 0.0165 (Behrens et al., 2007). The seeds and targets for tractography were the 104 cortical/subcortical regions (excluding cerebellum and brainstem regions) from multi-atlas parcellation, which were transformed into diffusion space using the inverse transform of the FA to T1 affine registration. The number of streamlines reaching each target region was divided by the total number of streamlines generated from the seed region and multiplied by average distance of streamlines to account for distance correction and ensure all connectivity values were between 0 and 1. This resulted in a 104 × 104 structural connectivity matrix that was used in network analyses described below.

Processed 18F-Flortaucipir PET images with uniform 8 mm full-width-at-half-maximum resolution were downloaded from the ADNI Archive (“Coreg, Avg, Std Img and Vox Size, Uniform Resolution”). An in-house processing pipeline was implemented to replicate these processing steps performed in ADNI (C. A. Brown et al., 2025). PET data were registered to T1-weighted MRI using ANTs rigid-body registration. An inferior cerebellar cortex reference region was used to generate SUVR maps and mean SUVR was extracted from all cortical/subcortical regions (excluding other cerebellar and brainstem regions). Aβ PET scans were used to determine positivity by clinical read (ABC) or Centiloid cutoff (ADNI).

### 2.4 Identification of tau epicenters

**Fig. 1.**
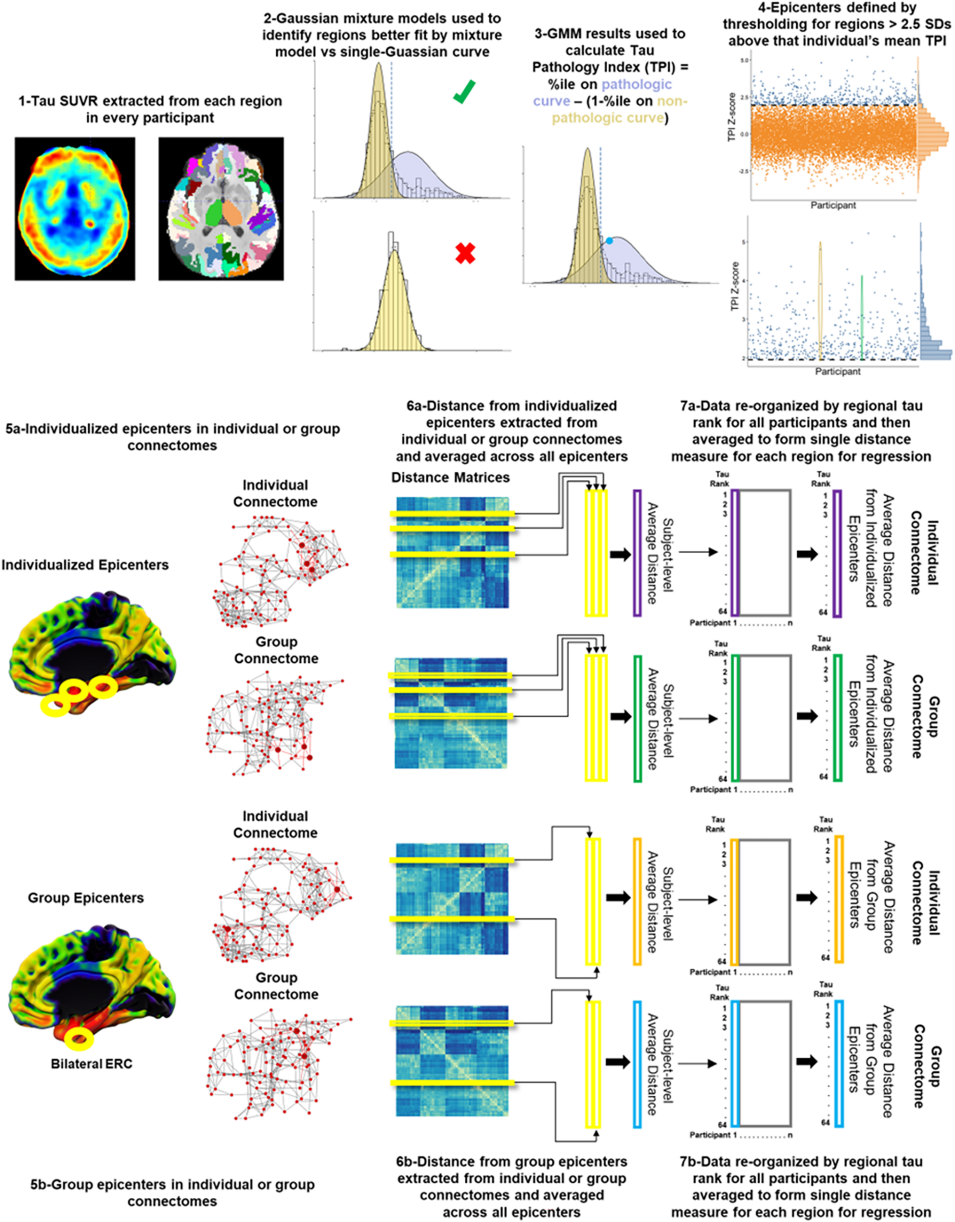
Schematic of overall methods. 1: Tau standardized uptake value ratio map (left) and cortical/subcortical parcellation of T1-weighted images using ANTs joint-label fusion (right). 2: Distribution of tau SUVR across all participants is fit with a single and two-Gaussian model. Regions that are better fit with a single-Gaussian model are not included in the analysis. 3: Gaussian-mixture model (GMM) results are used to identify pathologic (purple) and non-pathologic (orange) components and tau pathology index (TPI) is calculated from the relative position of SUVR values on these curves. An illustration of this calculation is provided for the point along the x-axis with position on each curve shown by the dashed line. 4: TPI scores are Z-scored within each participant and plotted with participant on the x-axis and TPI Z-score in each region on the y-axis. Regions with Z > 1.645 (purple) are considered epicenters (zoomed-in view shown in lower panel) with ovals highlighting the variable number of epicenters per participant. 5a/b: Individualized (a) or group (b) epicenters (black circles) are shown on the brain with color scale representing TPI. Epicenters are highlighted as larger nodes within individual and group connectomes (right). 6a/b: Distance matrices from network analyses are used to extract distance form each epicenter and then averaged to form a subject-level average distance from epicenter for each region using each of the four different approaches. 7a/b: The subject-level average distances are re-arranged in order of regional TPI rank to allow for alignment across participants and then averaged to form a group-level average distance from epicenters for each region using the four different approaches. These group-level averages are used as the distances for statistical analyses.

As ^18^F-flortaucipir has off-target binding, particularly in the basal ganglia, thalamus, and choroid plexus, SUVR data were converted to tau pathology index (TPI) using Gaussian-mixture models from a larger dataset combining all ADNI and ABC participants with available ^18^F-flortaucipir data (C. A. Brown et al., 2025). Briefly, data from all participants were examined in each ROI and fit with single Gaussian and two-Gaussian models to identify regions with only non-specific binding. Regions that were better fit by the single than 2-Gaussian model were excluded from further PET analyses leading to the exclusion of 20 bilateral region pairs (40 regions). The 2-Gaussian model was extracted from the remaining 64 regions with the component with the higher mean (i.e., right-shifted) considered the pathologic distribution and the component with the lower mean (i.e., left-shifted) considered the non-pathologic distribution (Fig. 1). TPI was then calculated using the following equation:

$$\begin{array}{l} \left(1\right)\;TPI=Percentile\;on\;Pathologic\;Component\\ \;\;\;\;\;-\left(1-Percentile\;on\;Non\;-\;Pathologic\;Component\right). \end{array}$$

See figures 1 and 2 from Brown et al. (2025) for additional details regarding TPI calculation and patterns across disease stages.

**Fig. 2.**
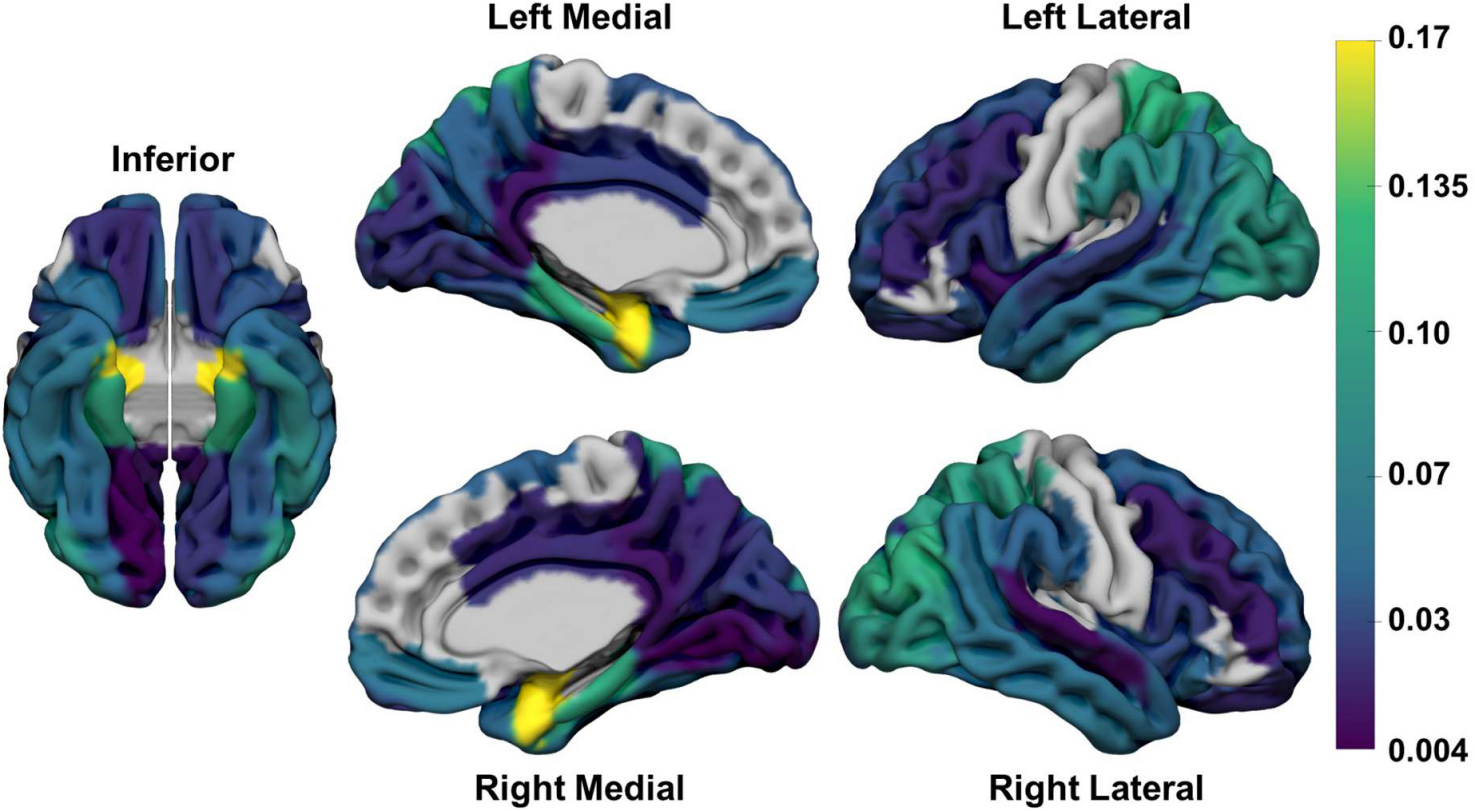
Frequency of epicenters. The locations of epicenters are shown on an MNI brain surface. The inferior surface (left) and left (top) and right (bottom) medial and lateral surfaces are shown. The color scale represents the proportion of participants for which each region was an epicenter. Regions with no color overlay reflect those that were better fit by the single Gaussian model.

The TPI scores across all regions were then Z-scored within each participant, and individualized epicenters were defined as regions with Z > 1.645 (Fig. 1). This outlier-detection approach allows for individuals to have a variable number of epicenters, rather than assigning the same number of epicenters to all individuals as has been often done previously (Franzmeier, Dewenter, et al., 2020; Franzmeier, Neitzel, et al., 2020; Steward et al., 2023). Moreover, it identifies areas that have greater tau than other regions within the same individual, which can be thought of as a likely epicenter from where tau may be spreading, rather than simply applying a cutoff across individuals. By using this approach, further individualization is possible as higher number of epicenters can be used in those with more widespread/multi-focal tau, while single epicenters can be used in those with more focal accumulation. Sensitivity analyses tested separate thresholds for defining individual epicenters (see Supplementary Material). A canonical group epicenter, left and right ERC, was also selected based on prior work and known susceptibility of this region in early AD (Ravikumar et al., 2024; Yushkevich et al., 2021).

### 2.5 Structural connectome development and analysis

Structural connectomes were formed by thresholding the connectivity matrices output from probabilistic tractography to exclude the bottom 10% of edges. This thresholded adjacency matrix was input to the R *iGraph* package to form an undirected weighted edge network. Connectivity strengths were inversed to form costs prior to implementing the Dijkstra algorithm to convert the network to a distance matrix of shortest paths between nodes. A separate group connectome was formed by averaging the connectivity matrices from all CU Aβ− participants prior to thresholding and performing network analysis as described for individual connectomes. Sensitivity analyses tested different thresholds for connectivity matrices ranging from excluding the bottom 0–80% of edges, and the distance matrices were compared for equivalence. Structural connectivity distance from tau epicenters was calculated by averaging the distance of each region from all tau epicenters. Separate averages were calculated for individualized and group structural connectomes, as well as for individualized and canonical epicenters.

### 2.6 Statistical analyses

R version 4.4.1 was used for all statistical analyses with the following packages: tidyverse, dplyr, broom, naniar, lme4, rstatix, cocorr, lm.beta, ggplot2, ggpubr, and ggExtra. We first evaluated for study (ADNI vs. ABC) effects to determine whether additional harmonization steps were necessary. There were no effects of study on the average distance from tau epicenter (*p* > 0.56, *η*^2^ < 0.01, average TPI (p = 0.073, *η^2^* = 0.03), or number of epicenters (*p* = 0.331, *η^2^* = 0.013) after accounting for amyloid status. Therefore, no additional harmonization steps, such as NeuroComBat, were applied beyond the harmonized data processing performed on these scans with highly overlapping acquisition parameters.

Prior to statistical analyses, data for each participant were re-organized by regional tau burden, such that each participant’s data were ordered from highest TPI region to lowest TPI region. Average distance from epicenter was then calculated for each of the 64 regions by averaging data across all participants within each training-test split. This resulted in four different values for each region: (1) average distance from individualized epicenters using individual connectomes (IE-IC), (2) average distance from individualized epicenters using group connectome (IE-GC), (3) average distance from canonical group epicenters using individual connectomes (GE-IC), and (4) average distance from canonical group epicenters using group connectome (GE-GC). Prior to calculating average TPI for each region, linear mixed effects models were used to regress out age, sex, education, race, time between tau PET and dMRI scan, and study (ABC or ADNI) with random effect of participant from TPI values. The resulting residual TPI values, representing TPI values adjusted for these covariates, were then averaged across all participants based on TPI rank.

Four linear regression models were tested in each training dataset using each of the average structural connectivity-based distances for each region as predictors of regional TPI. The resulting regression models were then used to predict regional TPI within each validation dataset. Regressions comparing the model-predicted TPI with measured TPI were used to assess goodness-of-fit in the validation cohorts. We compared the R^2^ between repeated folds for each model using paired t-tests to evaluate for significant differences between model performance. Analyses were then repeated by averaging values separately in Aβ+ and Aβ− individuals with the addition of a Distance × Aβ status interaction term to the regressions. Due to significant interactions, relationships within the Aβ+ and Aβ− groups were examined separately. Again, models were generated in each training dataset and applied to the corresponding dataset with 95% confidence intervals across train–test splits used to compare effect sizes between regression models.

We then applied the average regression model generated across all Aβ+ training splits at the individual participant level to predict regional TPI in each Aβ+ participant separately. Regressions comparing the predicted TPI with measured TPI were performed to assess the goodness-of-fit for each model within each participant. The standardized *β*-coefficient was extracted for each participant as a measure of effect size. Repeated measures ANOVA was used to test whether the four models differed in the average effect size across Aβ+ participants, and post hoc pairwise comparisons were performed using Bonferroni correction for multiple comparisons. Additional analyses explored factors contributing to performance at the individual level, including TPI in tau epicenters, number of epicenters, age, sex, and clinical severity.

Finally, we explored the association between structural connectivity distance from tau epicenters at baseline and longitudinal tau accumulation in a subset of Aβ+ participants with available longitudinal tau PET. Each model included the average distance from epicenters and baseline regional TPI as predictors and annualized change in TPI as the outcome. Due to the smaller sample size, all data were analyzed together without separate training/validation datasets. Hittner’s Z, a modification of Dunn and Clark’s Z, was used to compare the effect sizes of the distance terms in each of the four models (Hittner et al., 2003). We then applied each of these models generated at the group level to individual participant data and compared predicted annualized change in TPI with actual change in TPI using linear regression. The standardized *β*-coefficient was extracted from each participant as a measure of effect size. Repeated measures ANOVA was used to test whether the four models differed in the average effect sizes across participants, and post hoc pairwise comparisons were performed using Bonferroni correction for multiple comparisons.

## 3 Results

### 3.1 Participant characteristics

A total of 249 participants (93 ABC, 157 ADNI) with amyloid status, tau PET, and dMRI were initially identified for analyses. Quality control resulted in the exclusion of 7 (3 ABC, 4 ADNI) dMRI scans due to excessive motion or eddy current outliers, while 9 additional participants (8 ABC, 1 ADNI) were excluded from statistical analyses due to having 0 tau epicenters (see Sections 2.3 and 2.4. for details). A total of 233 participants were then randomly assigned to 5 separate testing folds with sizes ranging from 46 to 49 participants with the remaining participants used for training. A subset of 49 participants had longitudinal tau PET available with average 3.18 ± 1.35 years between scans. The sample demographics of the full dataset and longitudinal subset are given in Table 1.

### 3.2 Identification of tau epicenters

Regional TPI values were Z-scored within each participant to identify individualized epicenters, using a threshold of Z ≥ 1.645 (≥95%ile of values) as a cutoff (additional cutoffs were tested in sensitivity analyses, see below). This resulted in an average of 3.4 epicenters (median = 4, range = 1–7) being identified per included participant (9 participants excluded due to 0 epicenters). The distribution of epicenters is shown in Figure 2 and full list of epicenters is given in Supplementary Tables S1 and S2. The most frequent epicenters were left amygdala (n = 51), right ERC (n = 49), right amygdala (n = 48), and left ERC (n = 39). There was moderate overlap in epicenter distribution between folds (ICC = 0.65 [IQR: 0.51–0.75]), although there was strong overlap between each fold and the overall distribution of epicenters in the whole cohort (ICC = 0.84 [IQR: 0.86–0.89].

### 3.3 Individual epicenters and connectomes best explain cross-sectional regional tau burden

Subject-level average structural connectivity distances from tau epicenters and TPI were re-arranged for each participant based on the rank of TPI in each ROI prior to averaging across the entire group for each tau-ranked ROI (see Fig. 1). This allows for averaging across all participants despite different epicenters and regional tau burden, so that group-wide linear regression models can be performed. The average distance from tau epicenter was then used to explain regional tau burden using four different models in each training dataset: (1) a fully group-based model with canonical ERC epicenters and group connectome (GE-GC), (2) group canonical epicenters and individualized connectomes (GE-IC), (3) individualized epicenters and group connectome (IE-GC), and (4) a fully individualized model with individualized epicenters and connectomes (IE-IC). The results of all linear regressions are given in Table 2.

**Table 2.** Regression results.

	IE-IC	IE-GC	GE-IC	GE-GC	IEIC vs. IEGC	IEIC vs. GEIC	IEIC vs. GEGC
Training	*β* = -0.91 (0.012) *t* = -17.1 (<0.001)	*β* = -0.83 (0.027) *t* = -11.9 (<0.001)	*β* = -0.57 (0.054) *t* = -5.47 (<0.001)	*β* = -0.62 (0.026) *t* = -6.29 (<0.001)	*t* = 7.98 (0.001)	*t* = 17.2 (<0.001)	*t* = 35.5 (<0.001)
Testing	*β* = 0.86 (0.060) *t* = -17.1 (<0.001)	*β* = 0.57 (0.17) *t* = 5.89 (<0.001)	*β* = 0.35 (0.10) *t* = 2.95 (0.002)	*β* = 0.38 (0.026) *t* = 3.25 (0.004)	*t* = 3.79 (0.019)	*t* = 26.2 (<0.001)	*t* = 20.0 (<0.001)
Aβ+ Training	*β* = -0.93 (0.001) *t* = -20.4 (<0.001)	*β* = -0.59 (0.075) *t* = -5.85 (<0.001)	*β* = -0.72 (0.039) *t* = -8.19 (<0.001)	*β* = -0.64 (0.046) *t* = -6.67 (<0.001)	*t* = 10.1 (<0.001)	*t* = 12.4 (<0.001)	*t* = 13.9 (<0.001)
Aβ+ Testing	*β* = 0.85 (0.031) *t* = 12.8 (<0.001)	*β* = 0.30 (0.35) *t* = 2.66 (0.01)	*β* = 0.46 (0.080) *t* = 4.15 (<0.001)	*β* = 0.41 (0.13) *t* = 3.64 (<0.001)	*t* = 3.69 (0.021)	*t* = 9.60 (<0.001)	*t* = 8.19 (<0.001)
Aβ− Training	*β* = -0.81 (0.027) *t* = -11.1 (<0.001)	*β* = -0.76 (0.036) *t* = -9.33 (<0.001)	*β* = -0.21 (0.091) *t* = -1.67 (0.10)	*β* = -0.35 (0.069) *t* = -2.92 (0.005)	*t* = 3.13 (0.035)	*t* = 20.2 (<0.001)	*t* = 23.1 (<0.001)
Aβ− Testing	*β* = 0.73 (0.012) *t* = 9.22 (<0.001)	*β* = 0.51 (0.17) *t* = 4.98 (<0.001)	*β* = 0.13 (0.16) *t* = 1.03 (0.31)	*β* = 0.18 (0.16) *t* = 1.48 (0.014)	*t* = 2.65 (0.057)	*t* = 15.3 (<0.001)	*t* = 17.0 (<0.001)

The mean standardized regression coefficient (*β*) with (S.D.) across the five-folds along with average *t*-statistic and (*p*-value) for each model (*df* = 62). Comparison of the fully individualized model with all other models is shown in the final three columns with paired *t*-test (*p*-value) results across folds shown (*df* = 4). IE: individualized epicenters, IC: individual connectomes, GE: group epicenters, GC: group connectomes.

**Fig. 3.**
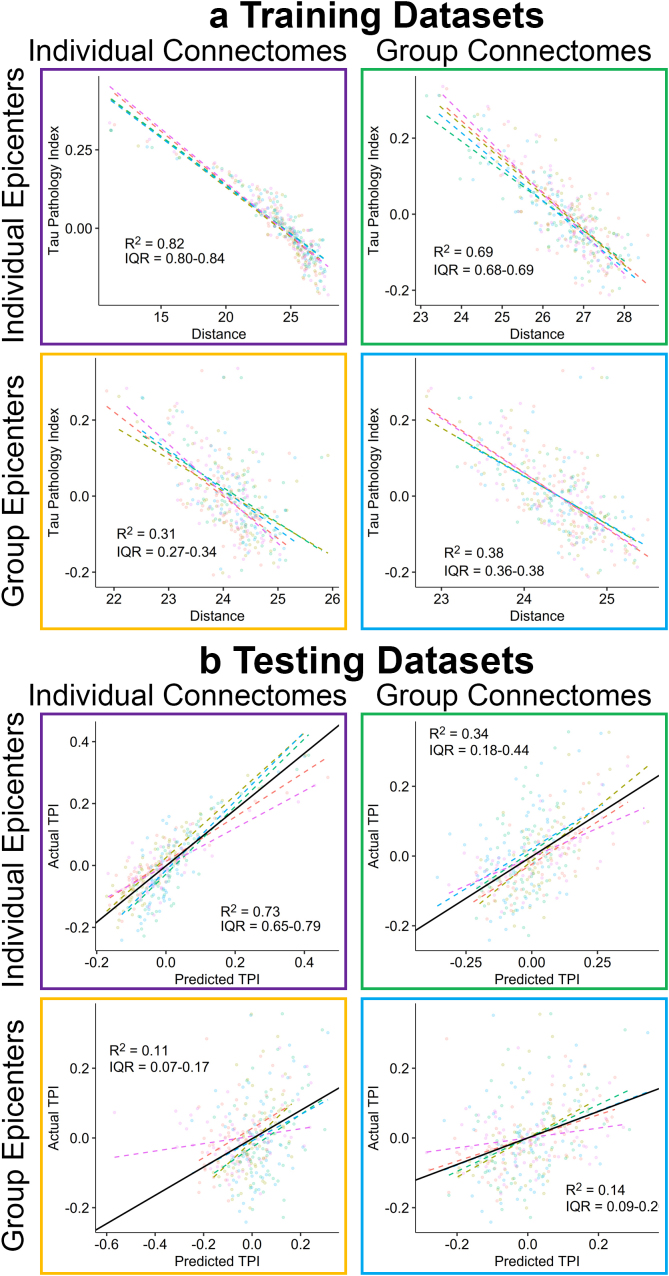
Prediction of regional TPI using structural connectivity distance. (a) Tau pathology index plotted against structural connectivity distance for each of the four models in the training datasets. (b) Measured TPI plotted against regional TPI obtained from applying the models in (a) to the validation datasets. (a–b) Each data point represents a brain region with coloring indicating the fold. Dashed lines represent the linear best-fit within each fold and the solid line (b) represents the average fit in the testing dataset. *R^2^* and interquartile range (IQR) are shown. IE: Individualized epicenters, GE: group epicenters, IC: individualized connectomes, GC: group connectome.

**Fig. 4.**
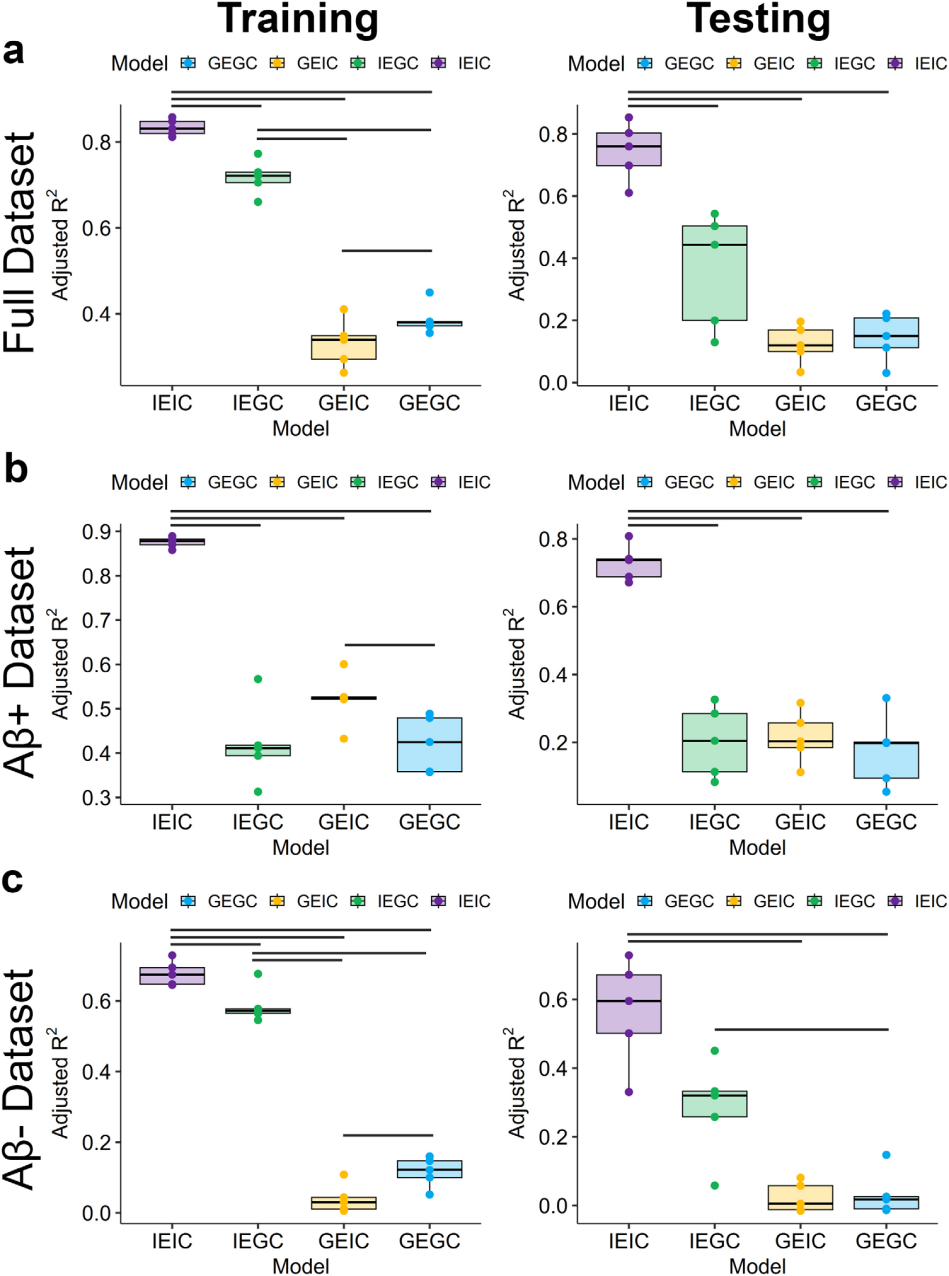
Model performance across folds. Adjusted *R*^2^ across folds is shown for each model in the full data set (a), Aβ+ dataset (b), and Aβ− dataset (c) with performance in the training set shown on top and application to testing datasets shown on bottom. Each point represents a fold. Significance bars indicate significant differences between model performance using paired t-tests.

Shorter connectivity distance was associated with higher regional TPI in all models (Fig. 3a): (1) GE-GC (*R^2^* = 0.39 [IQR: 0.37–0.38], *RMSE* = 0.081 [IQR: 0.080–0.082]), (2) GE-IC (*R^2^* = 0.33 [IQR: 0.29–0.35], *RMSE* = 0.085 [IQR: 0.084–0.088]), (3) IE-GC (*R^2^* = 0.72 [IQR: 0.71–0.73], *RMSE* = 0.055 [IQR: 0.053–0.057]), (4) IE-IC (*R^2^* = 0.83 [IQR: 0.82–0.85], *RMSE* = 0.042 [IQR: 0.041–0.045]). The resulting models were then applied to the testing dataset with significant association between model-predicted TPI and measured TPI for all models (Fig. 3b): (1) GE-GC (*R^2^* = 0.14 [IQR: 0.11–0.21], *RMSE* = 0.096 [IQR: 0.081–0.110]), (2) GE-IC (*R^2^* = 0.12 [IQR: 0.10–0.17], *RMSE* = 0.098 [IQR: 0.083–0.111]), (3) IE-GC (*R^2^* = 0.36 [IQR: 0.20–0.50], *RMSE* = 0.084 [IQR: 0.061–0.110]), and (4) IE-IC (*R^2^* = 0.75 [IQR: 0.70–0.80], *RMSE* = 0.052 [IQR: 0.042–0.058]). The fully individualized model had significantly greater association/prediction of regional tau burden than all other models in both the training (*t* ≥ 7.98, *p* ≤ 0.001) and testing (*t* ≥ 3.75, *p* ≤ 0.02) datasets (Fig. 4a).

### 3.4 Amyloid positivity strengthens the relationship between connectivity and tau burden

**Fig. 5.**
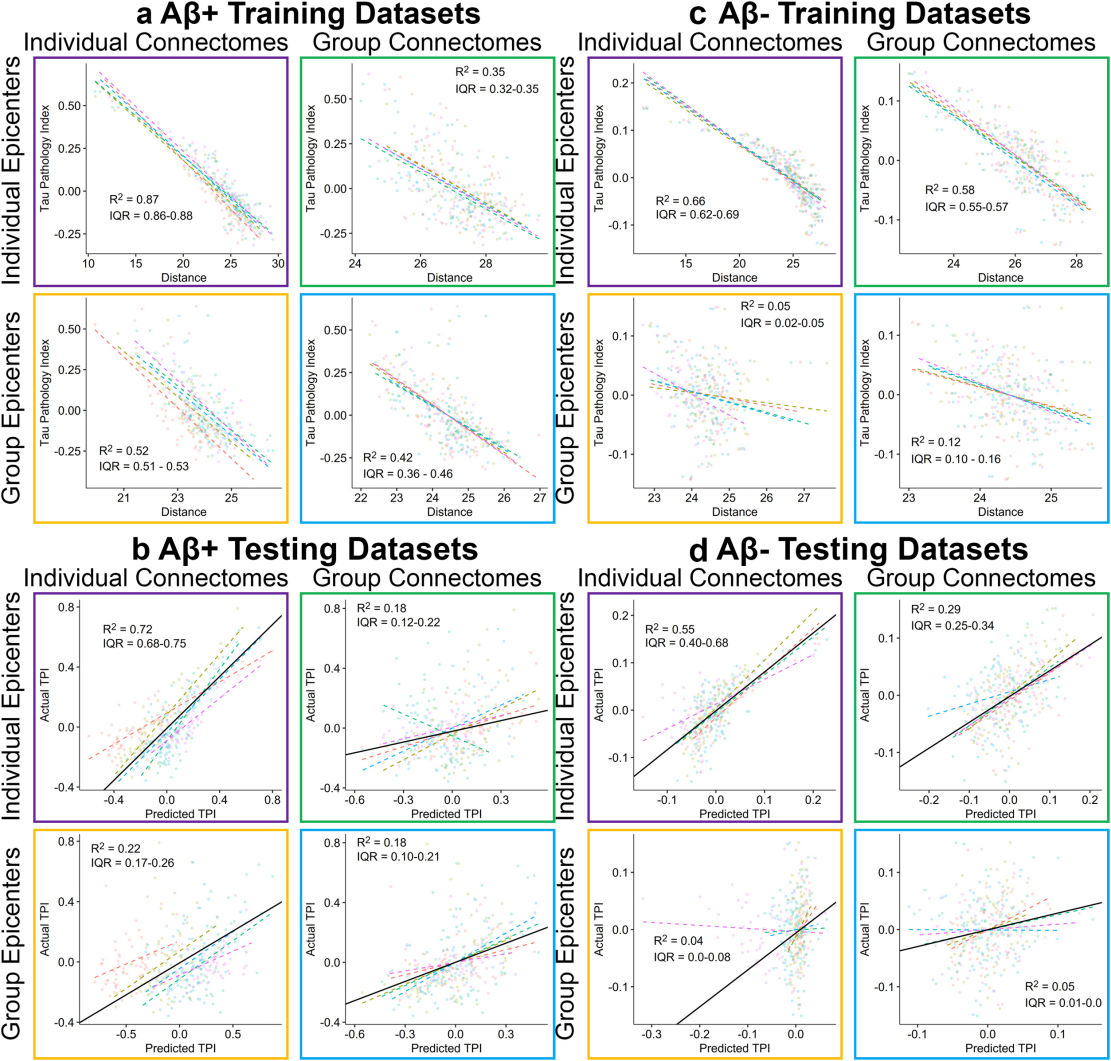
Prediction of regional TPI using structural connectivity distance in Aβ+ and Aβ− groups separately. Tau pathology index plotted against structural connectivity distance for each of the four models in the training cohort for Aβ+ (a) and Aβ− (c) groups. Measured TPI plotted against regional TPI obtained from applying the models in (a or c) to the validation datasets in Aβ+ (b) and Aβ− (d) groups. (a–d) Each data point represents a brain region, solid lines are the linear best-fit and red-dashed lines are the 95% prediction interval. *R^2^* and interquartile range (IQR) are shown. IE: Individualized epicenters, GE: group epicenters, IC: individualized connectomes, GC: group connectome.

We next re-organized data based on Aβ status to generate separate average regional TPIs and distances from tau epicenters for Aβ+ and Aβ− groups to evaluate for differences in model performance between groups (Table 2). Results of these models in the training datasets (Fig. 5a) demonstrated: (1) stronger association in Aβ+ (*R^2^* = 0.43 [IQR: 0.37–0.49], *RMSE* = 0.137 [IQR: 0.136–0.138]) than in Aβ− (*R^2^* = 0.13 [IQR: 0.11–0.16], *RMSE* = 0.052 [IQR: 0.051–0.052]) for the GE-GC model, (2) significant association in Aβ+ (*R^2^* = 0.53 [IQR: 0.53–0.53], *RMSE* = 0.125 [IQR: 0.119–0.130]) but not in Aβ− (*R^2^* = 0.05 [IQR: 0.03–0.06], *RMSE* = 0.054 [IQR: 0.0536–0.0547]) for the GE-IC model, (3) weaker association in Aβ+ (*R^2^* = 0.43 [IQR: 0.40–0.43], *RMSE* = 0.137 [IQR: 0.132–0.151]) than in Aβ− (*R^2^* = 0.59 [IQR: 0.57–0.58], *RMSE* = 0.035 [IQR: 0.0356–0.0368]) for the IE-GC model, and (4) stronger association in Aβ+ (*R^2^* = 0.88 [IQR: 0.87–0.88], *RMSE* = 0.064 [IQR: 0.060–0.064]) than in Aβ− (*R^2^* = 0.68 [IQR: 0.65–0.70], *RMSE* = 0.031 [IQR: 0.0303–0.0326]) for the IE-IC model. The fully individualized model had significantly greater association with regional tau burden than with all other models in both the Aβ+ group (*t* ≥ 9.32, *p* < 0.001) and Aβ− group (*t* ≥ 3.58, *p* ≤ 0.023) in the Aβ− group (Fig. 4b, c).

We then applied these separate models for the Aβ+ and Aβ− groups to their respective testing datasets (Table 2). Results demonstrated that all models predicted regional TPI in Aβ+ individuals but only the individualized epicenter models predicted TPI in Aβ− individuals (Fig. 5b): (1) GE-GC (Aβ+: *R^2^* = 0.19 [IQR: 0.11–0.21], *RMSE* = 0.167 [IQR: 0.142–0.191]; Aβ−: *R^2^* = 0.05 [IQR: 0.01–0.04], *RMSE* = 0.054 [IQR: 0.053–0.057]), (2) GE-IC (Aβ+: *R^2^* = 0.23 [IQR: 0.20–0.27], *RMSE* = 0.164 [IQR: 0.141–0.201]; Aβ−: *R^2^* = 0.04 [IQR: 0.0–0.07], *RMSE* = 0.055 [IQR: 0.053–0.057]), (3) IE-GC (Aβ+: *R^2^* = 0.22 [IQR: 0.13–0.30], *RMSE* = 0.166 [IQR: 0.123–0.198]; Aβ−: *R^2^* = 0.30 [IQR: 0.27–0.34], *RMSE* = 0.047 [IQR: 0.043–0.049]), and (4) IE-IC (Aβ+: *R^2^* = 0.73 [IQR: 0.69–0.75], *RMSE* = 0.096 [IQR: 0.083–0.113]; Aβ−: *R^2^* = 0.57 [IQR: 0.51–0.68], *RMSE* = 0.036 [IQR: 0.033–0.041]). The fully individualized model provided significantly greater prediction of regional tau burden than all other models in the Aβ+ group (*t* ≥ 3.37, *p* ≤ 0.028) and compared with the GE-GC and GE-IC models (*t* ≥ 14.8, *p* < 0.001) but not the IE-GC model (*t* = 2.57, *p* = 0.062) in the Aβ− group (Fig. 4b, c).

### 3.5 Fully individualized models provide improved prediction of subject-level tau patterns

**Fig. 6.**
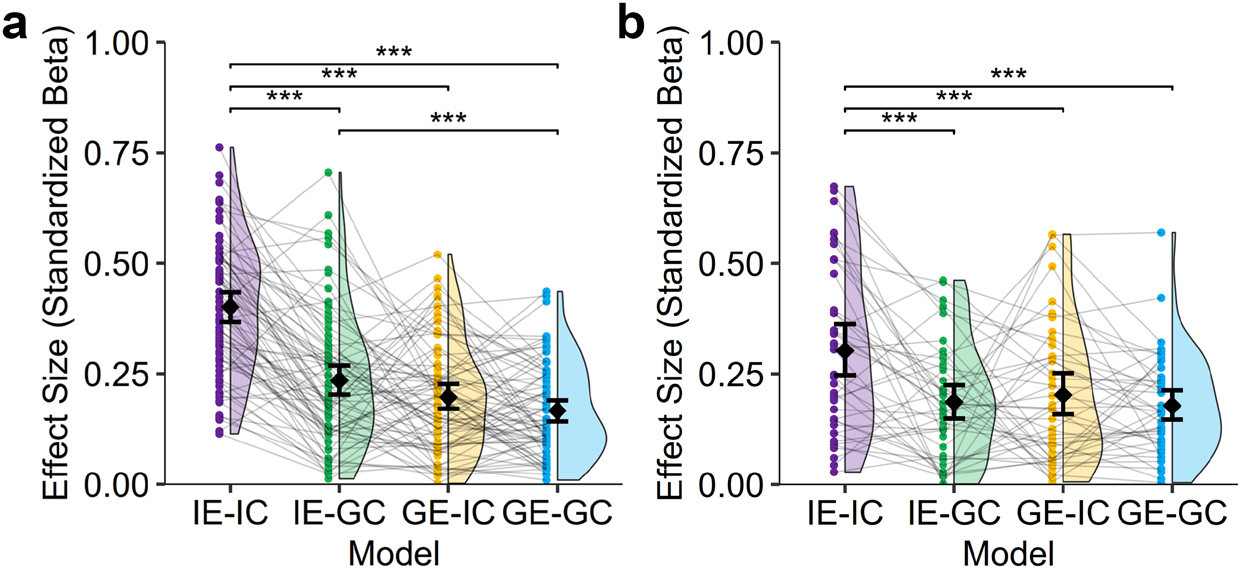
Subject-level prediction of regional TPI. Differences in effect size when applying each model to individual participant cross-sectional (a) and longitudinal (b) data in Aβ+ participants. Each data point represents an individual participant with half-violin plots showing the distribution of data and lines connecting data points from the same participant. Diamonds indicate the group mean standardized *β*-value, and error bars are for the 95% confidence interval for the mean. Differences were examined using post hoc paired *t*-tests with Bonferroni correction, ***p < 0.0001. IE: individual epicenters, GE: group epicenters, IC: individual connectome, GC: group connectome.

We next applied the Aβ+ models generated above to the Aβ+ participants at the subject level to predict regional tau burden. Next, a regression was run to compare the predicted TPI in each region with the measured TPI, and the standardized β coefficient was extracted as a measure of effect size (Fig. 6a). Repeated-measures ANOVA found that there was a significant effect of model on the mean effect size (*F_3,225_* = 73.0, *p* < 0.001). Post hoc paired *t-*tests demonstrated that effect size of fully individualized IE-IC model (mean *β_Std_* = 0.40 ± 0.15) was significantly higher than the IE-GC (mean *β_Std_* = 0.24 ± 0.15), GE-IC (mean *β_Std_* = 0.20 ± 0.12), and GE-GC (mean *β_Std_* = 0.17 ± 0.11) models (*t* ≥ 9.36, *p* < 0.001). The IE-GC model also had significantly stronger effect size than the GE-GC model (*t* = 3.81, *p* = 0.002), but there were no other significant differences between models. We next examined factors that influenced model performance and found that there was no significant effect of age, sex, or education for any model. For the IE-IC model, higher performance was associated with more tau epicenters (*β_Std_* = 0.25 [0.03–0.47], *t*(70) = 2.30, *p* = 0.024) and higher TPI in epicenters (*β_Std_* = 0.38 [0.16–0.60], *t*(70) = 3.39, *p* = 0.001). There was no association between model performance and epicenter number or TPI in epicenters for the other models.

### 3.6 Individualized models explain longitudinal change in regional tau burden

**Fig. 7.**
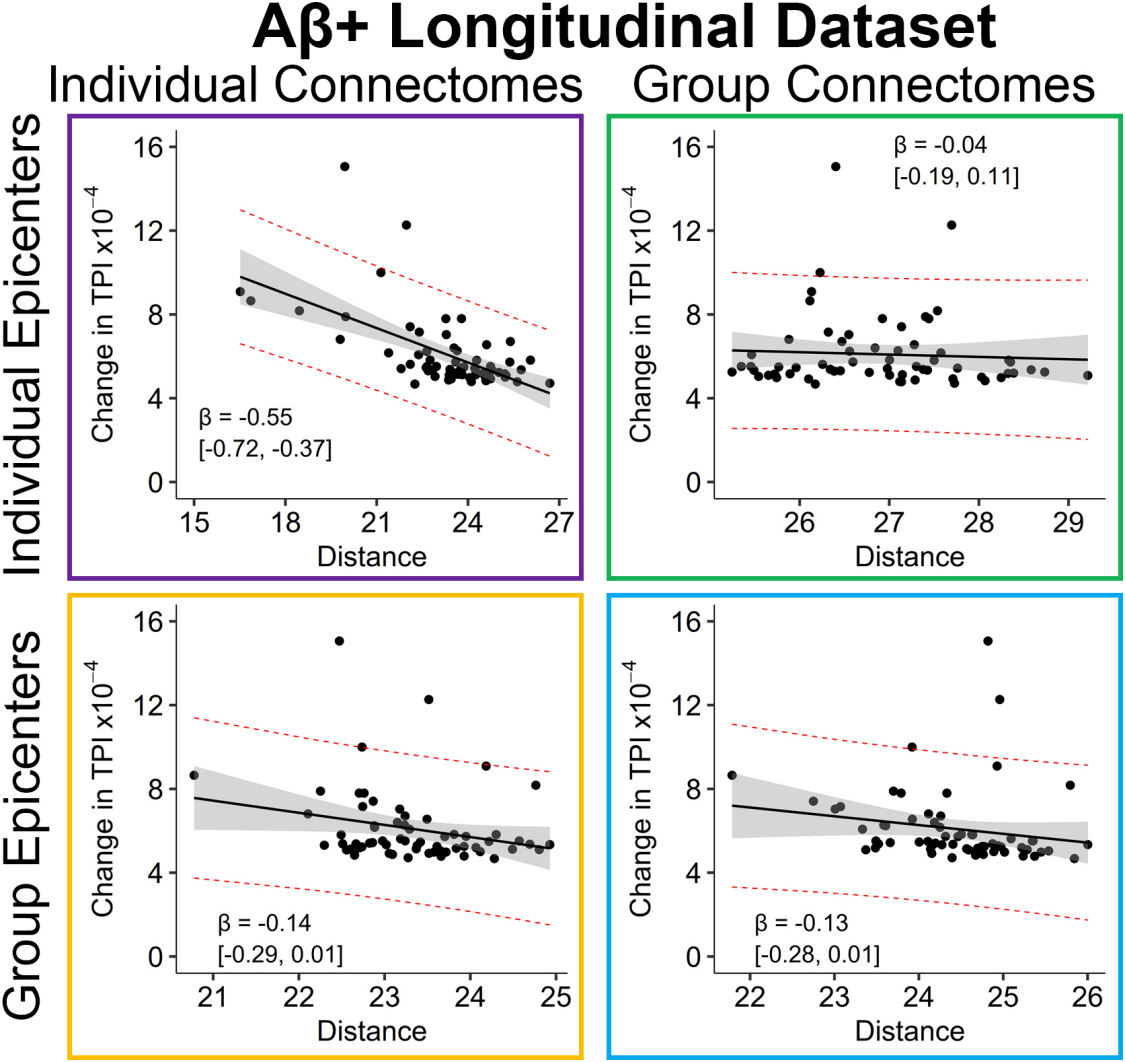
Association between longitudinal change in TPI and structural connectivity distance. Annualized change in tau pathology index (x10^-4^) after controlling for baseline TPI for each of the four models in the Aβ+ longitudinal cohort. Each data point represents a brain region, solid lines are the linear best-fit, and red-dashed lines are the 95% prediction interval. The standardized *β* [95% CI] is shown. IE: individual epicenters, GE: group epicenters, IC: individual connectome, GC: group connectome.

Finally, we explored whether each of the models could explain longitudinal change in regional tau burden within a subset of 49 Aβ+ participants with longitudinal data available. There were 21 CU, 21 MCI, and 7 AD patients with longitudinal data available. Given the small sample size, the full dataset was used rather than splitting into training and testing datasets. All models included regional baseline TPI as a covariate, average distance from epicenters at baseline as the predictor of interest, and annualized change in regional TPI as the outcome. All models explained longitudinal change in regional TPI, but distance from epicenters was only significant in the IE-IC and GE-GC models (Fig. 7): (1) GE-GC (*R^2^* = 0.77, *β_Std_* = -0.13 [-0.28, 0.01], *t* = -1.85, *p* = 0.07), (2) GE-IC (*R^2^* = 0.77, *β_Std_* = -0.14 [-0.29, 0.01], *t* = -1.89, *p* = 0.063), (3) IE-GC (*R^2^* = 0.76, *β_Std_* = -0.04 [-0.19, 0.11], *t* = -0.52, *p* = 0.61), (4) IE-IC (*R^2^* = 0.85, *β_Std_* = -0.55 [-0.72, -0.37], *t* = -6.28, *p* < 0.001). The fully individualized model had significantly stronger association with annualized change in TPI than all other models (*Hittner’s Z* ≥ 2.89, *p* ≤ 0.004).

We applied these models to predict subject-level change in regional TPI after controlling for baseline TPI and extracted the standardized beta values for each model as measure of effect size (Fig. 6b). Repeated-measures ANOVA found that there was a significant effect of model of the mean effect size (*F_3,126_* = 10.4, *p* < 0.001). Post hoc paired *t*-tests demonstrated that effect size of the fully individualized IE-IC model (mean *β_Std_* = 0.30 ± 0.19) was significantly higher than that of the IE-GC (mean *β_Std_* = 0.19 ± 0.13), GE-IC (mean *β_Std_* = 0.20 ± 0.16), and GE-GC (mean *β_Std_* = 0.18 ± 0.11) models (*t* ≥ 4.16, *p* ≤ 0.001). There were no other significant differences between the other models.

### 3.7 Sensitivity analyses

As several methodological aspects could influence the results of the study, we carried out several sensitivity analyses. First, we tested alternative thresholds for the connectomes ranging from the top 20 to 100% of connections included. The resulting distances were >98.5% identical for individual and group connectomes, indicating essentially no impact of the threshold selection on results. Next, we repeated analyses using alternative thresholds for defining individualized epicenters with higher thresholds of *Z* > 1.96 and *Z* > 2.32. Increasing the threshold resulted in exclusion of more participants with a greater proportion of CU participants excluded with increasing the threshold. The results of analyses were largely similar when testing alternative thresholds (Supplementary Tables S3–S5; Supplementary Figs. S1 and S2).

## 4 Discussion

In this study, we used a fully individualized approach based on structural connectivity and epicenters of tau pathology to predict regional tau burden in older adults on the AD continuum. This model explained >80% of the variance in regional tau burden, outperforming models that incorporated group canonical epicenters and/or group connectomes with higher variance explained and improved prediction in the validation dataset. We show that incorporating individualized epicenters lead to significant explanation of regional tau burden in both Aβ+ and Aβ− individuals, but combining individualized epicenters with individualized connectomes produces the strongest explanation in Aβ+ participants. Similarly, the fully individualized model outperformed all other models when predicting regional tau burden at the single-subject level in Aβ+ participants both cross-sectionally and longitudinally. Overall, these findings provide strong support for network-based propagation of tau pathology and demonstrate the power of a fully individualized approach to overcome limitations of current population-based approaches.

We first sought to identify individualized epicenters of tau pathology to better capture heterogeneity of tau patterns across individuals. Similar to prior studies that have identified patient-centered epicenters of pathology, the most common epicenters of tau pathology were bilateral amygdala and ERC (Franzmeier, Dewenter, et al., 2020; Vogel et al., 2020). These areas are known to be early sites of tau neurofibrillary tangle (NFT) deposition using quantitative post-mortem analyses and represent early Braak stage regions (Ravikumar et al., 2024; Yushkevich et al., 2021). While the hippocampus (and particularly anterior hippocampus) is also an early site of tau deposition, its proximity to choroid plexus limits the ability to detect pathologic binding of ^18^F-flortaucipir *in vivo* (Johnson et al., 2016; Lee et al., 2018). Beyond these typical epicenters, we did see large heterogeneity in regions identified as tau epicenters although with some posterior predominance, and future studies should further explore the underlying factors leading to this variability. Of note, the epicenter approach used here highlights areas from which tau may be spreading, but does not necessarily imply that it is an initial site of tau deposition. Overall, the patterns of tau epicenters, as well as regions excluded due to lack of specific binding, closely follow regions known to be involved in AD and those typically spared, respectively (Brier et al., 2016; Xia et al., 2017).

We next constructed individualized connectomes to further test our hypothesis that fully individualized approaches will improve prediction of regional tau burden. While most studies using connectivity to predict tau spread have relied on functional connectivity, functional connectomes are subject to significant noise at the individual level and only recently has it been possible to reproduce consistently high-quality fMRI data (Hermosillo et al., 2024). Therefore, we opted to use structural connectomes based on probabilistic tractography from dMRI, which is amenable to construction of individual connectomes (Bonilha et al., 2015; Buchanan et al., 2014). One potential pitfall when using either functional or structural connectomes is determining a threshold of connectivity to include as an edge in a connectome (Tsai, 2018; Zhang et al., 2022). While this remains an open area of debate, using a weighted-edge algorithm based on structural connectivity resulted in highly reproducible distance between nodes across multiple thresholds for edge existence (ranging from retaining all edges to only the top 20%) with >98% equivalence.

To compare our data across individuals with varying epicenters, we reorganized each participant’s data based on regional burden similar to prior studies (Franzmeier, Dewenter, et al., 2020). Using this approach, we reproduced results from prior studies that have used functional connectomes, demonstrating higher regional tau burden is associated with shorter structural connectivity-based distance from tau epicenters when using either canonical or individualized epicenters with group connectomes. Effect sizes of these group connectome models were slightly lower than previously published functional connectome-based studies with standardized *β*-values close to 0.64–0.72 in the present study compared with 0.65–0.85 in prior studies (Franzmeier, Dewenter, et al., 2020; Franzmeier et al., 2019; Thompson et al., 2024; Vogel et al., 2020). This may be due to subtle analytic differences including the smaller number of anatomic regions used in the present study compared with the highly parcellated Schaeffer atlas typically used in functional studies (Franzmeier, Dewenter, et al., 2020; Franzmeier et al., 2019), using regional tau burden quantity here compared with ordinal rank (Franzmeier, Dewenter, et al., 2020), and/or using CU Aβ− older adults as opposed to large number of healthy younger adults for generation of a group connectome (Franzmeier, Dewenter, et al., 2020; Jacobs et al., 2018; Vogel et al., 2020).

Importantly, we build upon these prior studies by using fully individualized models with both individual epicenters *and* individual connectomes. As has been previously hypothesized, individualized connectomes improved prediction at both the group level and subject level (Vogel et al., 2023). However, combining individual epicenters and connectomes in a fully individualized model provided the largest explanation for regional tau burden when statistically comparing effect sizes of different models. This was the case in both training and test datasets across the whole cohort and when restricting analyses to Aβ+ participants. Moreover, the fully individualized model outperformed all other models when predicting regional tau burden at the subject level with nearly 70% improvement compared with the next best model.

Similar to studies using group epicenters and/or connectomes, we were able to replicate our findings longitudinally in a subset of Aβ+ participants with shorter connectivity distance to tau epicenters at baseline associated with greater longitudinal tau accumulation when using either the fully population-based or individualized models (Franzmeier, Dewenter, et al., 2020; Franzmeier, Neitzel, et al., 2020; Leuzy et al., 2023). Due to the already small sample size, we did not restrict our analyses to epicenters meeting a specific criterion for tau positivity as has often been done in prior studies (Franzmeier, Dewenter, et al., 2020). Nevertheless, we found that the fully individualized approach provided the strongest prediction of longitudinal change in regional tau burden both at the group level and individual subject level. Our results add to these prior studies in providing strong support that regions that are more closely connected to tau epicenters accumulate tau at a faster rate than more distantly connected regions.

While prior studies using group-defined epicenters and connectomes could suggest either selective vulnerability of networks and/or cell-to-cell transmission of tau pathology, we believe that our findings further enhance support for a network topography of tau spread, and that cell-to-cell transmission is likely a mechanism for this network vulnerability. This is best reflected by the large heterogeneity of epicenters in our study, spanning across multiple networks, as well as the improvement seen when moving from a group connectome to individual connectome. The fully individualized approach allows for both variability in epicenters, network organization, and strength of connections at the individual level. Moreover, structural connectomes more closely measure physical connections between brain regions and are dominated by monosynaptic connections, thus aligning more closely with the concept of transsynaptic spread. While interindividual variability in selectively vulnerable networks and in specific regions within those networks that are most vulnerable could potentially explain our findings, more likely this result supports the mechanism of transsynaptic spread of tau pathology which converges with data from mouse and iPSC models. Consistent with this, simply providing distance along an individual’s structural connectome from their individual epicenters in an anatomic/network agnostic approach led to robust prediction of regional tau burden that outperformed other models both cross-sectionally and longitudinally.

It should be noted that while we focused on subject-level prediction in Aβ+ individuals, the individual epicenter models predict regional tau burden in Aβ− individuals as well. This is consistent with prior work using functional connectomes (Franzmeier et al., 2019; Vogel et al., 2020). The most common epicenters in Aβ− individuals remained the amygdala and ERC. These regions are common locations of tau in primary age-related tauopathy (PART), which may in part drive the effects in Aβ− individuals (Crary et al., 2014). Consistent with this, there is evidence that tau pathology also demonstrates cell-to-cell transmission in PART (Kaufman et al., 2018). However, we did also observe tau epicenters in Aβ− participants outside of the temporal lobe in regions that would not be expected to represent PART, and the sensitivity of ^18^F-flortaucipir to tau in PART remains controversial (Ghirelli et al., 2020; Josephs et al., 2024; Lowe et al., 2020). Therefore, this pattern of ^18^F-flortaucipir uptake among connected regions could also represent some non-specific signal of neurodegeneration, such as iron deposition or co-pathology (Choi et al., 2018; Marquié et al., 2015). Other neuropathologic proteins, including Aβ and α-synuclein, have also been shown to have cell-to-cell transmission in model systems (Cirrito et al., 2005; Lazarov et al., 2002; Luk et al., 2012). Further work and comparison of these relationships when using different tracers will be important to better understand this phenomenon.

There are several key limitations to the present study. First, the dataset is primarily CU or MCI with smaller number of individuals with dementia level impairment and is made up of mostly late-onset cases of AD. Future studies of populations should be conducted to further assess whether these findings generalize to other presentations of AD. Second, the sample size for longitudinal analyses was relatively small, not allowing for separation of discovery and validation datasets. Future work will be necessary to replicate these findings in other longitudinal cohorts with available high-quality dMRI and tau PET. Finally, we focused on only structural connectomes, which may ignore relevant data from functional connectomes and other network types. Recent work has sought to combine multiple modalities using cortical gradients or multi-modal connectome analyses to improve prediction by leveraging different information provided from various modalities (Ottoy et al., 2024; Thompson et al., 2024). Future work will need to evaluate the influence of combining these modalities, but is somewhat limited by the dearth of high-quality fMRI data that can be reproduced at the single-subject level.

The study has several important strengths. First, the sensitivity analyses and successful application to validation datasets indicate that these findings are robust to methodological choices and not the result of over-fitting. Second, the inclusion of the ABC cohort in this study led to improved representation of historically underrepresented populations. Much of the prior work focusing on connectivity-based tau spread has been in ADNI (which is also used in the present study) and BIOFINDER, which have limited representation of non-white groups (Franzmeier, Neitzel, et al., 2020; Raman et al., 2022; Vogel et al., 2020; Weiner et al., 2023). Together, these features indicate that our findings should be reproducible in other cohorts. Lastly, we demonstrate that a fully individualized approach is superior to population-based methods at both the group and individual level cross-sectionally and longitudinally, the latter of which has important implications for using similar techniques to monitor disease progression and treatment effects.

In conclusion, we used a fully individualized approach to model regional tau burden based on structural connectivity with epicenters of tau pathology. This approach accounted for more than 80% of the variance in regional tau burden and outperformed models that incorporated group epicenters and/or group connectomes. This remained true when restricting analyses to Aβ+ participants, as well as when applying models to individual participant data. Moreover, the fully individualized approach was the best predictor of longitudinal change in regional tau burden at both the group level and subject level. Together, these findings highlight the ability of a fully individualized approach to improve predictive modeling at the group level and single-subject level, as well as provide strongest *in vivo* evidence to-date supporting the notion of cell-to-cell transmission of tau pathology in human disease.

## Supplementary Material

Supplementary Material

## Data Availability

All requests for raw and analyzed data from the ABC cohort will be reviewed by the Penn Neurodegenerative Data Sharing Committee (PNDSC) and shared for appropriate uses through a data-sharing agreement (https://www.pennbindlab.com/data-sharing). Anonymized data from ABC will be shared upon request to the corresponding author by a qualified academic investigator for the purpose of replicating procedures and results in this article. Data are not publicly available due to privacy protections outlined in the participant informed consent. Documents related to study protocols, informed consent, and other documentation can similarly be made available upon request. All ADNI data are shared without embargo through the LONI Image and Data Archive (https://ida.loni.usc.edu/), a secure research data repository. Interested scientists may obtain access to ADNI imaging, clinical, genomic, and biomarker data for the purposes of scientific investigation, teaching, or planning clinical research studies. Access is contingent on adherence to the ADNI Data Use Agreement and the publications’ policies (https://adni.loni.usc.edu/data-samples/adni-data/#AccessData). All code (imaging processing scripts, R scripts) used in these analyses is available upon request to the corresponding author.
