# Supplementary material for: Fully individualized models for cross-sectional and longitudinal network-based tau spread
Source: Imaging Neurosci (Camb). 2025 Dec 9;3:IMAG.a.1053. doi: 10.1162/IMAG.a.1053 (PMC12690294; doi:10.1162/IMAG.a.1053)
Supplement: Supplementary Material [file IMAG.a.1053_supp.pdf]

Supplemental Table 1. Epicenters by Amyloid Status

Region	Amyloid Positive	Amyloid Negative	Total
Left Amygdala	34	17	51
Right Entorhinal area	35	14	49
Right Amygdala	31	17	48
Left Entorhinal area	27	12	39
Right Parahippocampal gyrus	21	11	32
Right Superior occipital gyrus	8	16	24
Left Calcarine cortex	3	20	23
Left Parahippocampal gyrus	12	11	23
Right Calcarine cortex	2	20	22
Left Superior occipital gyrus	7	13	20
Left Cuneus	1	18	19
Left Medial frontal cortex	2	17	19
Right Medial frontal cortex	2	17	19
Right Cuneus	4	15	19
Right Gyrus rectus	1	16	17
Left Gyrus rectus	3	14	17
Left Inferior occipital gyrus	7	9	16
Right Superior frontal gyrus	5	10	15
Right Inferior temporal gyrus	13	1	14
Left Inferior temporal gyrus	8	5	13
Right Inferior occipital gyrus	8	5	13
Right Middle occipital gyrus	5	7	12
Left Superior frontal gyrus	3	8	11
Right Middle temporal gyrus	7	4	11
Left Anterior orbital gyrus	2	8	10
Right Posterior orbital gyrus	2	8	10
Left Superior parietal lobule	5	5	10
Right Middle cingulate gyrus	1	8	9
Right Anterior orbital gyrus	2	7	9
Left Angular gyrus	4	5	9
Left Middle occipital gyrus	4	5	9
Left Middle temporal gyrus	8	1	9
Right Anterior cingulate gyrus	0	8	8
Right Superior parietal lobule	3	5	8
Left Posterior cingulate gyrus	4	4	8
Right Occipital fusiform gyrus	5	3	8
Right Angular gyrus	7	1	8
Right Fusiform gyrus	7	1	8
Left Medial orbital gyrus	0	7	7
Left Opercular part of the inferior frontal gyrus	0	7	7
Left Anterior cingulate gyrus	1	6	7
Left Posterior orbital gyrus	1	6	7

Right Frontal operculum	3	4	7
Left Occipital fusiform gyrus	4	3	7
Left Temporal pole	6	1	7
Right Medial orbital gyrus	0	6	6
Left Fusiform gyrus	5	0	5
Left Middle cingulate gyrus	0	5	5
Left Anterior insula	1	4	5
Left Frontal operculum	1	4	5
Left Lingual gyrus	1	4	5
Right Anterior insula	1	4	5
Right Opercular part of the inferior frontal gyrus	1	4	5
Left Superior temporal gyrus	4	0	4
Left Middle frontal gyrus	0	4	4
Right Temporal pole	1	3	4
Right Supramarginal gyrus	3	1	4
Right Posterior cingulate gyrus	0	3	3
Left Precuneus	2	0	2
Right Superior temporal gyrus	2	0	2
Left Supramarginal gyrus	0	2	2
Right Lingual gyrus	0	2	2
Right Precuneus	0	1	1

Epicenter count within Aβ+, Aβ-, and all participants with color shading reflecting magnitude within each column

Supplemental Table 2. Epicenters by Fold

Region	Fold 1	Fold 2	Fold 3	Fold 4	Fold 5	Total
Left Amygdala	6	9	9	11	16	51
Right Entorhinal area	4	11	9	12	13	49
Right Amygdala	5	9	10	13	11	48
Left Entorhinal area	6	7	8	6	12	39
Right Parahippocampal gyrus	5	4	7	10	6	32
Right Superior occipital gyrus	4	5	8	5	2	24
Left Calcarine cortex	8	2	3	3	7	23
Left Parahippocampal gyrus	5	3	4	6	5	23
Right Calcarine cortex	6	6	4	1	5	22
Left Superior occipital gyrus	5	4	4	6	1	20
Left Cuneus	6	3	2	4	4	19
Left Medial frontal cortex	5	7	3	2	2	19
Right Cuneus	7	4	3	2	3	19
Right Medial frontal cortex	4	4	3	4	4	19
Left Gyrus rectus	4	6	2	1	4	17
Right Gyrus rectus	4	4	4	3	2	17
Left Inferior occipital gyrus	3	3	4	4	2	16
Right Inferior temporal gyrus	4	2	5	4	0	15
Right Superior frontal gyrus	1	1	2	4	7	15
Left Inferior temporal gyrus	3	1	4	3	3	14
Right Inferior occipital gyrus	1	3	5	3	1	13
Right Middle occipital gyrus	3	3	4	2	0	12
Right Middle temporal gyrus	3	2	2	2	3	12
Left Superior frontal gyrus	0	3	3	2	3	11
Left Anterior orbital gyrus	2	1	1	3	3	10
Left Angular gyrus	1	2	1	2	4	10
Left Middle occipital gyrus	1	2	4	3	0	10
Left Middle temporal gyrus	1	1	2	3	3	10
Left Superior parietal lobule	2	3	1	2	2	10
Right Posterior orbital gyrus	3	3	1	2	1	10
Right Anterior orbital gyrus	0	2	2	3	3	10
Right Middle cingulate gyrus	1	3	0	1	4	9
Left Posterior cingulate gyrus	1	3	0	2	2	8
Right Anterior cingulate gyrus	1	2	3	1	1	8
Right Angular gyrus	3	2	1	0	2	8
Right Fusiform gyrus	2	2	2	1	1	8
Right Occipital fusiform gyrus	1	1	4	2	0	8
Right Superior parietal lobule	3	2	2	0	1	8
Left Anterior cingulate gyrus	1	5	1	0	0	7
Left Medial orbital gyrus	2	2	1	2	0	7
Left Occipital fusiform gyrus	3	1	1	1	1	7
Left Opercular part of the inferior frontal gyrus	2	1	1	2	1	7

Left Posterior orbital gyrus	2	2	1	1	1	7
Left Temporal pole	2	1	1	3	0	7
Right Frontal operculum	4	0	2	0	1	7
Left Fusiform gyrus	1	0	2	3	0	6
Right Medial orbital gyrus	2	2	0	0	2	6
Left Frontal operculum	4	0	0	0	1	5
Left Lingual gyrus	3	0	2	0	0	5
Left Middle cingulate gyrus	1	2	1	0	1	5
Right Anterior insula	2	1	1	0	1	5
Right Opercular part of the inferior frontal gyrus	2	0	1	2	0	5
Left Anterior insula	0	0	1	0	4	5
Right Supramarginal gyrus	1	0	0	1	2	4
Right Temporal pole	1	0	2	1	0	4
Left Middle frontal gyrus	0	2	1	0	1	4
Left Superior temporal gyrus	0	0	1	2	1	4
Left Supramarginal gyrus	1	1	1	0	0	3
Right Posterior cingulate gyrus	0	2	1	0	0	3
Right Superior temporal gyrus	1	1	0	0	0	2
Left Precuneus	0	1	0	0	1	2
Right Lingual gyrus	0	1	1	0	0	2
Right Precuneus	0	0	1	0	0	1

Epicenter count within each fold and across all participants with color shading reflecting magnitude across folds and separately for across all participants.

Table S3. Demographics of Alternative Epicenter Models

	Z = 1.645 (n = 233)	Z = 1.96 (n = 205)	Z = 2.32 (n = 148)
**Age (range)**	71 (50-90)	71 (50-90)	71 (56-90)
**Sex**			
F	140 (60%)	124 (60%)	89 (60%)
M	93 (40%)	81 (40%)	59 (40%)
**Race**			
Asian	3 (1%)	3 (1%)	2 (1%)
Black	44 (19%)	38 (19%)	26 (18%)
Multiple	3 (1%)	3 (1%)	3 (2%)
White	183 (79%)	161 (79%)	117 (79%)
**Education (range)**	16 (9-20)	16 (9-20)	16 (9-20)
**Diagnosis**			
CU	160 (69%)	139 (68%)	93 (63%)
MCI	62 (27%)	56 (27%)	45 (30%)
Dementia	10 (4%)	10 (5%)	10 (7%)
**Amyloid Positive (%)**	85/233 (36%)	76/205 (37%)	65/148 (44%)
**Study**			
ABC	82 (35%)	65 (32%)	39 (26%)
ADNI	151 (65%)	140 (68%)	109 (74%)

Mean (range) for continuous measures and n (%) for categorical measures. F: Female, M: Male, CU: Cognitively unimpaired, MCI: Mild Cognitive Impairment

Table S4. Model performance using epicenter threshold of Z > 1.96

	IE-IC	IE-GC	GE-IC	GE-GC	IEIC vs IEGC	IEIC vs GEIC	IEIC vs GEGC
Training	β = -0.88 (0.011) t = -14.5, p < 0.001	β = -0.85 (0.015) t = -12.6, p < 0.001	β = -0.55 (0.048) t = -5.19, p < 0.001	β = -0.64 (0.053) t = -6.57, p < 0.001	t = 8.78, p < 0.001	t = 14.6, p < 0.001	t = 8.52, p < 0.001
Testing	β = 0.82 (0.043) t = 11.6, p < 0.001	β = 0.61 (0.096) t = 6.26, p < 0.001	β = 0.34 (0.13) t = 2.90, p = 0.005	β = 0.40 (0.14) t = 3.51, p < 0.001	t = 5.09, p = 0.007	t = 7.14 p = 0.002	t = 5.47, p = 0.005
Aβ+ Training	β = -0.92 (0.006) t = -18.7, p < 0.001	β = -0.68 (0.038) t = -7.38, p < 0.001	β = -0.72 (0.021) t = -8.12, p < 0.001	β = -0.66 (0.032) t = -6.98, p < 0.001	t = 12.9, p < 0.001	t = 19.4, p < 0.001	t = 16.3, p < 0.001
Aβ+ Testing	β = 0.83 (0.056) t = 11.9, p < 0.001	β = 0.41 (0.11) t = 3.61, p < 0.001	β = 0.47 (0.073) t = 4.24, p < 0.001	β = 0.42 (0.12) t = 3.71, p < 0.001	t = 6.32, p = 0.003	t = 9.56 p < 0.001	t = 7.15, p = 0.002
Aβ- Training	β = -0.76 (0.023) t = -9.33, p < 0.001	β = -0.76 (0.025) t = -9.20, p < 0.001	β = -0.17 (0.08) t = -1.34, p = 0.19	β = -0.36 (0.052) t = -3.09, p = 0.003	t = 0.47, p = 0.66	t = 19.2, p < 0.001	t = 14.8, p < 0.001
Aβ- Testing	β = 0.68 (0.093) t = 7.57, p < 0.001	β = 0.51 (0.15) t = 4.91, p < 0.001	β = 0.10 (0.18) t = 0.84, p = 0.40	β = 0.19 (0.089) t = 1.51, p = 0.14	t = 3.56, p = 0.024	t = 7.76 p = 0.001	t = 7.91, p = 0.001

Mean (s.d.) of standardized β coefficients are shown along with the average t-statistic and corresponding p-value for each model (left). Comparison between IEIC model performance and other models using paired t-statistics (right). IE: Individualized epicenters, IC: Individualized connectomes, GE: Group epicenter, GC: Group connectome.

Table S5. Model performance using epicenter threshold of Z > 2.32

	IE-IC	IE-GC	GE-IC	GE-GC	IEIC vs IEGC	IEIC vs GEIC	IEIC vs GEGC
Training	β = -0.88 (0.008) t = -14.3, p < 0.001	β = -0.77 (0.042) t = -9.58, p < 0.001	β = -0.55 (0.041) t = -5.20, p < 0.001	β = -0.59 (0.010) t = -5.74, p < 0.001	t = 5.61, p = 0.005	t = 19.7, p < 0.001	t = 38.2, p < 0.001
Testing	β = 0.81 (0.051) t = 11.2, p < 0.001	β = 0.48 (0.184) t = 4.63, p < 0.001	β = 0.32 (0.10) t = 2.70, p = 0.009	β = 0.36 (0.079) t = 3.05, p = 0.003	t = 4.39, p = 0.012	t = 10.8 p < 0.001	t = 8.75, p < 0.001
Aβ+ Training	β = -0.91 (0.012) t = -17.2, p < 0.001	β = -0.65 (0.054) t = -6.78, p < 0.001	β = -0.69 (0.027) t = -7.43, p < 0.001	β = -0.65 (0.014) t = -6.79, p < 0.001	t = 9.75, p < 0.001	t = 17.2, p < 0.001	t = 24.1, p < 0.001
Aβ+ Testing	β = 0.80 (0.079) t = 10.9, p < 0.001	β = 0.36 (0.18) t = 3.26, p = 0.002	β = 0.44 (0.098) t = 3.91, p < 0.001	β = 0.42 (0.080) t = 3.72, p < 0.001	t = 4.84, p = 0.008	t = 6.70 p = 0.003	t = 6.50, p = 0.003
Aβ- Training	β = -0.74 (0.013) t = -8.67, p < 0.001	β = -0.63 (0.079) t = -6.52, p < 0.001	β = -0.06 (0.068) t = -0.47, p = 0.64	β = -0.25 (0.050) t = -2.06, p = 0.044	t = 3.39, p = 0.028	t = 22.0, p < 0.001	t = 25.5, p < 0.001
Aβ- Testing	β = 0.64 (0.024) t = 6.50, p < 0.001	β = 0.35 (0.31) t = 3.33, p = 0.001	β = -0.03 (0.13) t = -0.28, p = 0.79	β = 0.13 (0.11) t = 1.02, p = 0.31	t = 2.05, p = 0.11	t = 11.2 p < 0.001	t = 10.9, p < 0.001

Mean (s.d.) of standardized β coefficients are shown along with the average t-statistic and corresponding p-value for each model (left). Comparison between IEIC model performance and other models using paired t-statistics (right). IE: Individualized epicenters, IC: Individualized connectomes, GE: Group epicenter, GC: Group connectome.

Figure S1.

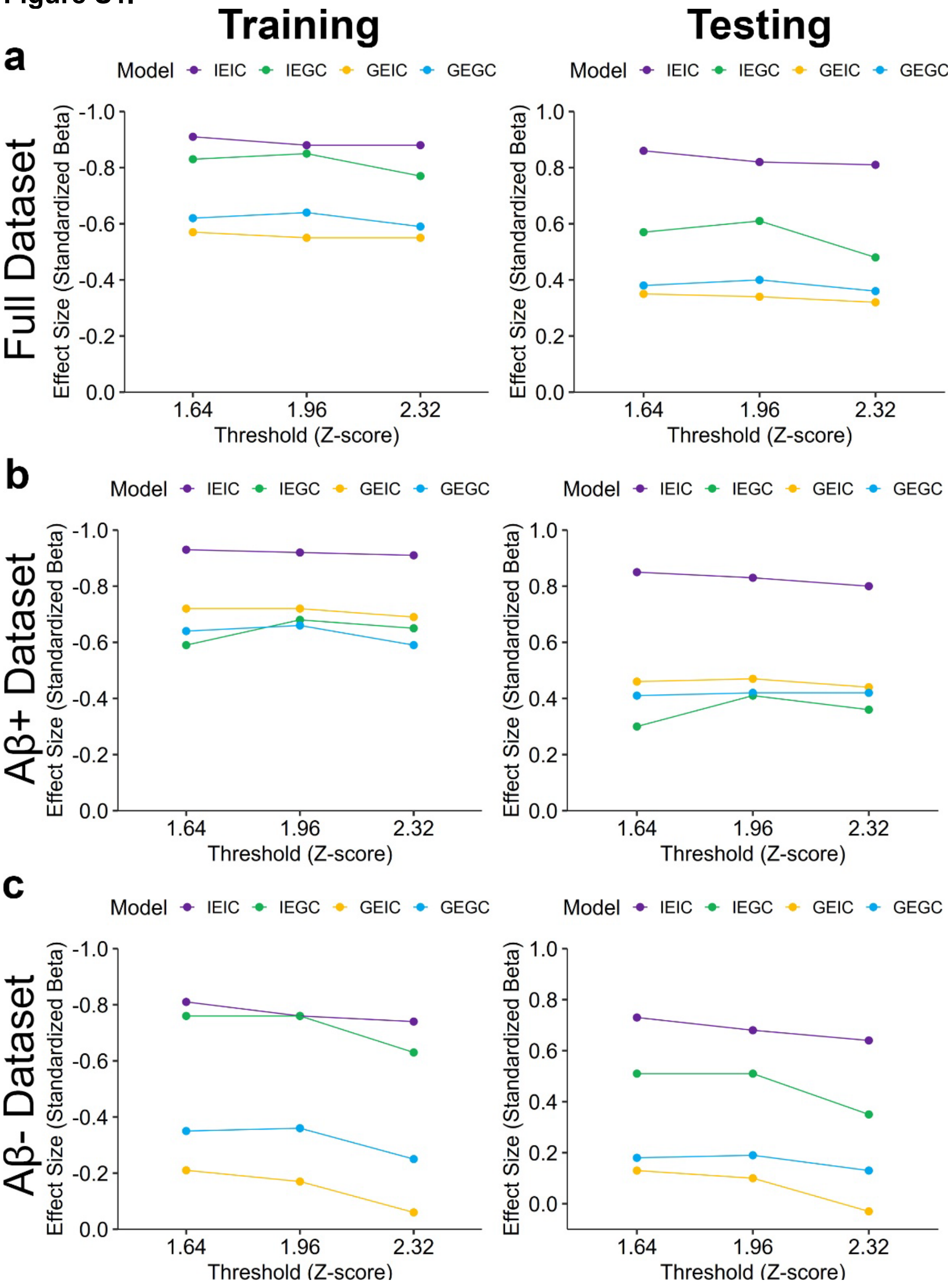

Figure S1. Effect size of models at various epicenter thresholds. Effect size of each model at different epicenter thresholds in the training (left) and testing (right) datasets across all participants (**a**), Aβ+ (**b**), and Aβ- (**c**) groups. Each point reflects the standardized β-value for the average Structural Connectivity Distance from Epicenter in the regression model predicting Regional Tau Pathology Index. More negative β-values reflect stronger effects on the left and more positive β-values reflect stronger effects on the right. The threshold of Z = 1.645 was used for the primary analyses reported in the main text. Performance in the training cohort was minimally impacted by increasing the threshold, but performance in the testing cohort showed a mild decline, which may in part be due to smaller sample size.

Figure S2.

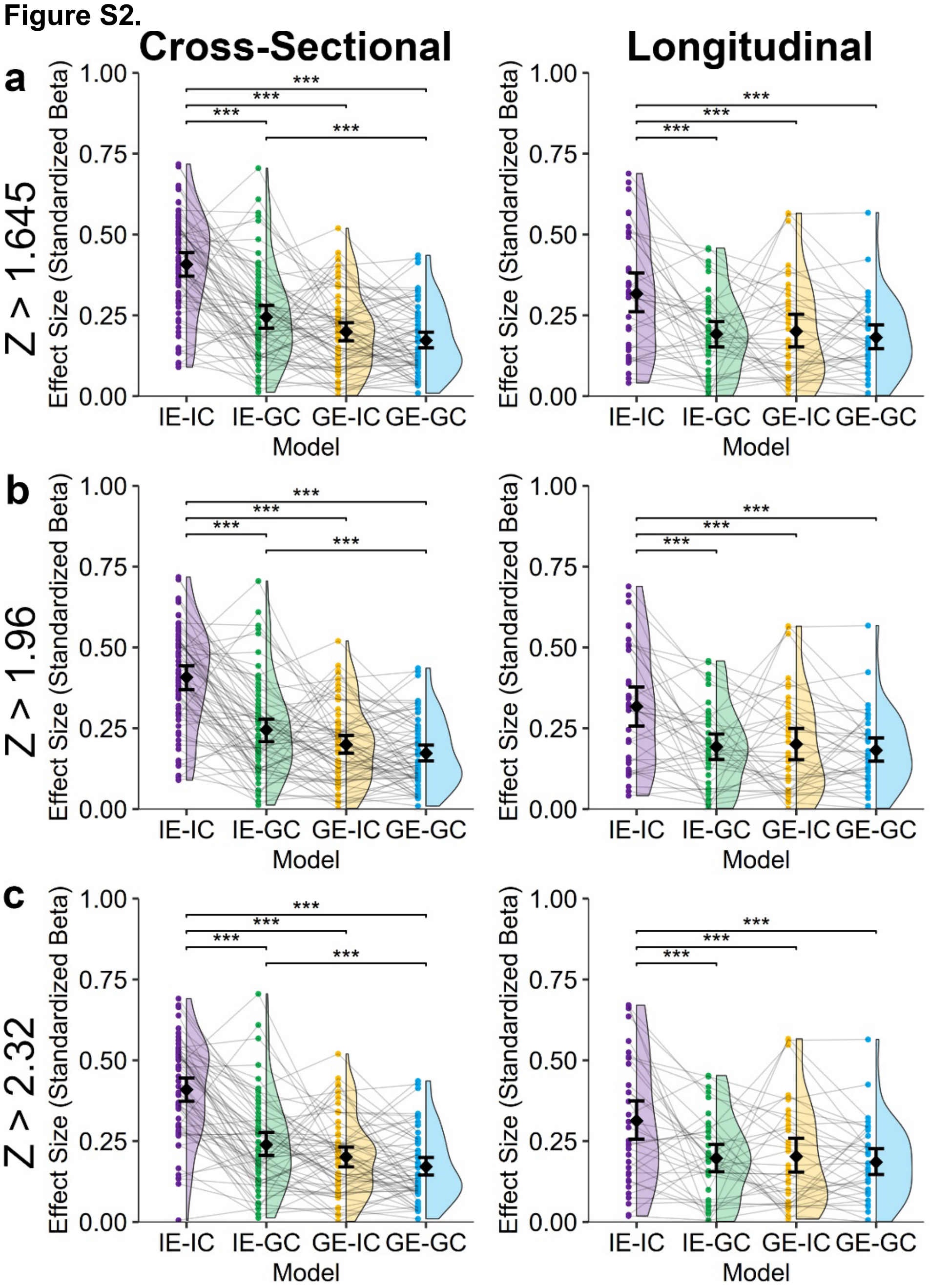

Figure S2. Subject-level prediction of regional TPI. Cross-sectional (left) and longitudinal (right) subject-level prediction of regional TPI using epicenter threshold of Z > 1.645 (**a**), Z > 1.96 (**b**), or Z > 2.32 (**c**) show similar effects. Lines connect individual participants. Diamonds indicate the group mean standardized β-value and error bars are for the 95% confidence interval for the mean. ***Bonferroni corrected $p < 0.0001$ on post-hoc *t*-test. IE: Individual epicenters, GE: Group epicenters, IC: Individual connectome, GC: Group connectome.
